# An innovative randomized response model based on a customizable random tool

**DOI:** 10.1371/journal.pone.0319780

**Published:** 2025-04-18

**Authors:** Ahmad M. Aboalkhair, Mohammad A. Zayed, Tamer Elbayoumi, Abdullah Alnefaie, Mahmaod Alrawad, Ahmed M. Elshehawey

**Affiliations:** 1 Department of Quantitative Methods, School of Business, King Faisal University, Al-Ahsa, Saudi Arabia; 2 Department of Applied Statistics and Insurance, Faculty of Commerce, Mansoura University, Mansoura, Egypt; 3 Department of Mathematics and Statistics, North Carolina A & T State University, Greensboro, North Carolina, United States of America; 4 College of Business Administration and Economics, Al-Hussein Bin Talal University, Ma’an, Jordan; 5 Department of Applied, Mathematical & Actuarial Statistics, Faculty of Commerce, Damietta University, New Damietta, Egypt; SKUMS: Shahrekord University of Medical Science, IRAN, ISLAMIC REPUBLIC OF

## Abstract

This paper suggests an innovative randomized response model utilizing customizable random tool. The suggested model offers a general framework for some previously pioneering randomized response models and generates new efficient models. Comparison of the efficiency between one of these newly generated models and other groundbreaking models through theoretical and numerical ways, demonstrates higher efficiency for the new generated model. Additionally, ethical considerations and privacy protection of the suggested model are examined.

## 1. Introduction

Sample surveys may present situations where individuals would rather withhold or provide false information about certain questions when dealing with an interviewer, like cases of drug use, psychiatric conditions, infidelity issues, delinquency, criminal abortion, illegitimacy details and even political party affiliation. Evasive response bias could indeed be challenging to evaluate. Warner [1] came up with a proposal that could help reduce this bias— and that is ensuring privacy for the interviewee by using randomized responses technique (RRT). In such setups, individuals are at liberty to keep their personal information private by giving responses in a random manner, which helps address the issue of evasive response bias.

The RRT in surveys aims to minimize or avoid response errors when questioning individuals about delicate topics. The basic idea behind a design of randomized response is that information will be collected indirectly from interviewees by asking questions whose answers cannot be known with certainty by an interviewer. Thus, in RRT usage it is believed that interviewees provide truthful information that can aid in estimation.

Even though Warner’s technique enables collecting answers on delicate matters while upholding anonymity, its estimations have a raised standard error because of utilizing the random tool. Following Warner’s initial suggestion, many researchers have expanded the RRT in different dimensions. Their focus, however, has always revolved around curtailing estimation variance and bolstering model efficiency; this they achieve through various means such as proposing parameters selection based on specific criteria aimed at minimizing variance and resorting to alternative estimation methods— but primarily by suggesting design modifications to Warner’s original model.

Design modification is the primary approach taken by the majority of studies to improve the efficiency of RRT. Several authors have suggested different modifications to Warner’s model with the goal of enhancing its efficacy [2–19].

Aboalkhair et al. [20] brought in an innovative effective model through a design modification approach based on three randomizing devices. In their work, Aboalkhair et al. [20] showed that their method is an efficient substitute for models introduced by Mangat & Singh and Warner. The study established that using a randomized multi-stage instrument, especially with a higher number of stages, raises the chance of the sensitive question being chosen without significantly impacting interviewee’ trust in the tool or their honesty hence, it led to the effectiveness of the RR technique is enhanced. The inspiration for this research comes from previous research, and its aim is to create a generalized randomized response model.

## 2. Previously pioneering models

### 2.1. Warner’s model

The groundbreaking RR model is suggested by Warner [1] to estimate the percentage of individuals who possess delicate attributes π. According to Warner’s model, the estimation of π with suitable changes of notation is:


$${\hat{\pi }}_{1}=\frac{\hat{\alpha }-\left(1-p_{1}\right)}{2p_{1}-1}$$ (1)

and variance given by:


$$V\left({\hat{\pi }}_{1}\right)=\frac{\text{π}\left(1-\text{π}\right)}{n}+\frac{p_{1}\left(1-p_{1}\right)}{n{\left[2p_{1}-1\right]}^{2}}$$ (2)

### 2.2. Mangat & Singh’s model

Mangat & Singh [5] introduced an efficient two-stage RR model. The estimate of π in Mangat & Singh’s design is:


$${\hat{\pi }}_{2}=\frac{\hat{\alpha }-\left(1-p_{2}\right)\left(1-p_{1}\right)}{2p_{1}-1+2p_{2}\left(1-p_{1}\right)}$$ (3)

with variance given by:


$$V\left({\hat{\pi }}_{2}\right)=\frac{\pi \left(1-\pi \right)}{n}+\frac{\left(1-p_{2}\right)\left(1-p_{1}\right)\left[1-\left(1-p_{2}\right)\left(1-p_{1}\right)\right]}{n{\left[2p_{1}-1+2p_{2}\left(1-p_{1}\right)\right]}^{2}}$$ (4)

Mangat & Singh [5] demonstrated that, their model outperforms the original Warner’s model by appropriately selecting any feasible values of $p_{1}$ and $p_{2}$.

### 2.3. Aboalkhair’s model

Aboalkhair [20] suggested an efficient model utilizing a three-stage random tool. Utilizing a random sample of n interviewees, Aboalkhair’s estimate of π and its variance with appropriate changes of notation are:


$${\hat{\pi }}_{3}=\left[\hat{\alpha }-q_{1}q_{2}q_{3}\right]{\left[1-2q_{1}q_{2}q_{3}\right]}^{-1},q_{1}q_{2}q_{3}\neq 0.5$$ (5)

and,


$$V\left({\hat{\pi }}_{3}\right)=[\text{π}\left(1-\text{π}\right)+q_{1}q_{2}q_{3}\left[1-q_{1}q_{2}q_{3}\right]{\left[1-2q_{1}q_{2}q_{3}\right]}^{-2}]/n$$ (6)

In the following section, we propose a generalized version of Aboalkhair’s model that incorporates previous models, such as that suggested by Mangat & Singh and Warner as special cases, from which new efficient RR models can be generated.

## 3. The suggested model

### 3.1. Model description

**Fig 1:**
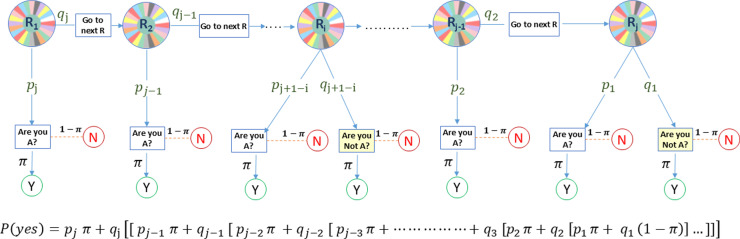
Suggested model flowchart.

To estimate *π* the proportion of individuals that have a delicate attribute (D) in specific population, each interviewee in a selected random sample is provided with a customizable random tool with j-stage as depicted in Fig 1. At the onset (in stage-one (S1)), the interviewee randomly chooses between two options: the first option being a yes/no query that determines if he/she has the delicate attribute, while the second option instructs them to proceed to the subsequent stage Si, (where i ranges from 2 to j-1). If he/she proceeds to (Si), he/she is given the same previous choice. If the interviewee reaches to the final stage (Sj), he/she is given a yes/no query about the sensitive attribute, similar to the original Warner’s model.

The probability of “Yes” ($\alpha_{j}$) will be:


$$\alpha_{j}=p_{j}\pi +q_{j}\left[p_{j-1}\pi +q_{j-1}\left[p_{j-2}\pi +q_{j-2}\left[p_{j-3}\pi +\ldots +q_{3}\left[p_{2}\pi +q_{2}\left[p_{1}\pi +q_{1}\left(1-\pi \right)\right]\right]\ldots \right]\right]\right],$$ (7)

where:

$p_{j+1-g}$: The probability of the question that determines if the interviewee has a specific delicate attribute or not shows up at stage g, as g =  1,2,3,…,j and $p_{j+1-g}+q_{j+1-g}=1$.

The estimator of *π* is as follows:


$${\hat{\text{π}}}_{j}=\left[{\hat{\alpha }}_{j}-Q_{j}\right]{\left[1-2Q_{j}\right]}^{-1},Qj\neq 0.5$$ (8)

where ${\hat{\alpha }}_{j}$ denotes the ratio of ‘yes’ answers in the sample and $Q_{j}=\prod_{\text{g}=1}^{\text{j}}q_{j+1-g}$.

Customize j=1 in Eq. (8) we get


$${\hat{\pi }}_{1}=\left[\hat{\alpha }-q_{1}\right]{\left[1-2q_{1}\right]}^{-1},q_{1}\neq 0.5$$

which coincides with Eq. (1). If we customize j=2, we get


$${\hat{\pi }}_{2}=\left[\hat{\alpha }-q_{1}q_{2}\right]{\left[1-2q_{1}q_{2}\right]}^{-1},q_{1}q_{2}\neq 0.5$$

which is coincide with Eq. (3) and if set j=3, we get Eq. (5).

### 3.2. Properties of the suggested estimator

Since $n{\hat{\alpha }}_{j}\sim Bin\left(n,\alpha \right)$, therefore ${\hat{\alpha }}_{j}$ is an unbiased estimator of *α*, and the variance of ${\hat{\text{π}}}_{j}$ is:

**Theorem 1.** *The proposed estimator variance is*


$$V\left({\hat{\pi }}_{j}\right)=[\pi \left(1-\pi \right)+Q_{j}\left[1-Q_{j}\right]{\left[1-2Q_{j}\right]}^{-2}]/n$$ (9)

**Proof.** Utilizing Eq. (8), $V\left({\hat{\pi }}_{j}\right)$ is


$$V\left({\hat{\pi }}_{j}\right)=\text{V}\left(\left[{\hat{\alpha }}_{j}-Q_{j}\right]{\left[1-2Q_{j}\right]}^{-1}\right)=\text{V}\left({\hat{\alpha }}_{j}\right){\left[1-2Q_{j}\right]}^{-2}$$ (10)

As $n{\hat{\alpha }}_{j}\sim Bin\left(n,\alpha \right)$, then


$$\text{V}\left({\hat{\alpha }}_{j}\right)=\alpha_{j}\left(1-\alpha_{j}\right)/n$$ (11)

Substitute by Eq. (11) in Eq. (10) then,


$$V\left({\hat{\pi }}_{j}\right)=\alpha_{j}\left(1-\alpha_{j}\right){\left[1-2Q_{j}\right]}^{-2}/n$$ (12)

Using Eq. (7), $\alpha_{j}\left(1-\alpha_{j}\right)$ can be calculated as,


$$\alpha_{j}\left(1-\alpha_{j}\right)=\text{π}\left(1-\text{π}\right){\left[1-2Q_{j}\right]}^{2}+Q_{j}\left[1-Q_{j}\right]$$ (13)

Then, Eq. (9) is obtained via substituting in Eq. (12) by Eq. (13).

Set j=1, in Eq. (9) we get


$$V\left({\hat{\pi }}_{1}\right)=[\text{π}\left(1-\text{π}\right)+q_{1}\left[1-q_{1}\right]{\left[1-2q_{1}\right]}^{-2}]/n$$

Which is coincide with Eq. (2), if we customize j=2, we get


$$V\left({\hat{\pi }}_{2}\right)=[\text{π}\left(1-\text{π}\right)+q_{1}q_{2}\left[1-q_{1}q_{2}\right]{\left[1-2q_{1}q_{2}\right]}^{-2}]/n$$

Which is coincide with Eq. (4) and if we customize j=3, we get Eq. (6).

**Theorem 2.** $V\left({\hat{\pi }}_{j}\right)$ *has an unbiased estimator given by*


$$\hat{V}\left({\hat{\text{π}}}_{\text{j}}\right)=\hat{\alpha_{j}}\left(1-\hat{\alpha_{j}}\right){\left[1-2Q_{j}\right]}^{-2}/\left(n-1\right)$$ (14)

**Proof.** The result holds by taking the expected value of Eq. (14).

### 3.3. Efficiency comparison of the suggested estimator

The proposed model with j-stage outperforms the model with j-g-stage, g=1.2…j−1 in terms of efficiency iff:


$$\overset{j}{\underset{i=j-g+1}{\prod }}q_{i}<\left[1-Q_{j-g}\right]{\left[1-Q_{j}\right]}^{-1}$$ (15)

## 4. Privacy protection

In the randomized response technique, all types of models are subject to several ethical considerations. The main purpose of them is to empower researchers to find the balance between their need to elicit sensitive information and the ethical treatment of participants. First, the respondents must voluntarily and explicitly agree to participate, being informed about the nature and manner of implementation of the RR technique, its purpose, and their freedom to refuse to take part. The researcher has to make the details of data gathering and publication evident by describing the purpose of the study and how it will be shared.

Furthermore, researchers need to consider the possible effects of asking sensitive questions on participants and try to minimize associated harm or distress resulting from responding to them. All studies using Randomized Response techniques have to receive ethical approval from institutional review boards or ethics committees so that research is conducted in line with the ethical standards giving consideration to protecting the rights and welfare of the research participants. Individual responses will be anonymous and untraceable, with a special focus on privacy protection for respondents.

### 4.1. Privacy protection measure

One of the fundamental aspects of the randomized response technique is preserving interviewee ‘s privacy. Several privacy measures are suggested by researchers such as Anderson [21], Lanke [22], Leysieffer and Warner [23], and Zhimin & Zaizai [24]. Based on the latter approach, the privacy measure for Warner’s model is:


$$M_{1}\left(R\right)=\frac{{\left(1-2q_{1}\right)}^{2}}{2q_{1}\left(1-q_{1}\right)}$$ (16)

Also, the measure of privacy protection for Mangat & Singh’s model can be expressed as follows:


$$M_{2}\left(R\right)=\frac{{\left(1-2q_{1}q_{2}\right)}^{2}}{2q_{1}q_{2}\left(1-q_{1}q_{2}\right)}$$ (17)

and for Aboalkhair’s model:


$$M_{3}\left(R\right)=\frac{{\left(1-2q_{1}q_{2}q_{3}\right)}^{2}}{2q_{1}q_{2}q_{3}\left(1-q_{1}q_{2}q_{3}\right)}$$ (18)

And for suggested model the design probabilities are:


$$P\left(yes\text{|}D\right)=1-Q_{j}\text{and}P(yes|\bar{D})=Q_{j}$$

$$P\left(no\text{|}D\right)=Q_{j}\;\text{and}P\left(no\text{|}\bar{D}\right)=1-Q_{j}$$

and


$$P\left(D\text{|}yes\right)=\frac{\pi }{\pi +\left(Q_{j}\right)\left(1-\pi \right)/\left(1-Q_{j}\right)}$$

$$P\left(D\text{|}no\right)=\frac{\pi }{\pi +\left(1-Q_{j}\right)\left(1-\pi \right)/\left(Q_{j}\right)}$$

Then, the privacy protection measure is:


$$M_{j}\left(R\right)=\left|1-\frac{1}{2}\left\{\tau \left(y\right)+\tau \left(n\right)\right\}\right|$$

where


$$\tau \left(y\right)=\frac{1-Q_{j}}{Q},\tau \left(n\right)=\frac{Q_{j}}{1-Q_{j}}$$

$$M_{j}\left(R\right)=\frac{{\left(1-2Q_{j}\right)}^{2}}{2Q_{j}\left(1-Q_{j}\right)}$$ (19)

To verify the validity of Eq. (19), set j = 1, j = 2 and j = 3 the measure of protection for Warner’s estimate, Mangat & Singh’s estimate and Aboalkhair’s estimate are obtained, as indicated by Eq. (16), Eq. (17), and Eq. (18) respectively.

A correlation between the privacy protection measure discussed earlier and the efficiency of each of the four models can be established. These correlation relationships are outlined as follows:


$$V\left({\hat{\text{π}}}_{1}\right)=\frac{\pi \left(1-\pi \right)}{n}+\frac{1}{2nM_{1}\left(R\right)}$$ (20)

$$V\left({\hat{\text{π}}}_{2}\right)=\frac{\pi \left(1-\pi \right)}{n}+\frac{1}{2nM_{2}\left(R\right)}$$ (21)

$$V\left({\hat{\text{π}}}_{3}\right)=\frac{\pi \left(1-\pi \right)}{n}+\frac{1}{2nM_{3}\left(R\right)}$$ (22)

$$V\left({\hat{\text{π}}}_{\text{j}}\right)=\frac{\pi \left(1-\pi \right)}{n}+\frac{1}{2nM_{j}\left(R\right)}$$ (23)

It is clear from Eqs (20–23) that as the values of $M_{j}\left(R\right)$ decrease, the efficiency of ${\hat{\pi }}_{j}$ also decreases. Moreover, Zhimin & Zaizai [24] demonstrated that a higher level of privacy protection for interviewees is achieved when their measure of privacy protection has smaller values. A balance act is required.

## 5. Suggested RR model with four-stage random tool

To get a particular meaning for the suggested model with j-stage random tool, we consider the scenario where the number of stages is customized to be four (j = 4). In the initial stage (S1), the interviewee chooses between two options randomly: the first option being a yes/no query that determines if he/she has the delicate attribute, while the second option instructs them to proceed to the subsequent stage Si, (Si, where i takes values of 2 and 3), he/she is given the same choice as in the first stage. If the interviewee reaches the final stage (S4), he/she is given a yes/no query about the sensitive attribute, similar to the original Warner’s model.

The probability of receiving a “Yes” response (*α*) can be:


$$\alpha_{4}=p_{4}\pi +q_{4}\left[p_{3}\pi +q_{3}\left[p_{2}\pi +q_{2}\left[p_{1}\pi +q_{1}\left(1-\pi \right)\right]\right]\right],$$ (24)

where:

$p_{5-s}$: The probability of the question that determines if the interviewee has a delicate attribute shows up at stage g, as s =  1,2,3,4 and $p_{5-s}+q_{5-s}=1$.

The estimator suggested for $\left({\hat{\pi }}_{4}\right)$ is:


$${\hat{\text{π}}}_{4}=\left[{\hat{\alpha }}_{4}-q_{1}q_{2}q_{3}q_{4}\right]{\left[1-2q_{1}q_{2}q_{3}q_{4}\right]}^{-1},q_{1}q_{2}q_{3}q_{4}\neq 0.5$$ (25)

where ${\hat{\alpha }}_{4}$ is the proportion of ‘yes’ answer in the sample.

### 5.1. Properties of estimator

***Corollary 1*.** *The proposed estimator variance* $V\left({\hat{\pi }}_{4}\right)$ *is*


$$V\left({\hat{\pi }}_{4}\right)=\pi \left(1-\pi \right)/n+q_{1}q_{2}q_{3}q_{4}\left[1-q_{1}q_{2}q_{3}q_{4}\right]{\left[1-2q_{1}q_{2}q_{3}q_{4}\right]}^{-2}/n$$ (26)

***Corollary 2.*** $V\left({\hat{\pi }}_{j}\right)$ *has an unbiased estimator given by*


$$\hat{V}\left({\hat{\pi }}_{4}\right)=\hat{\alpha_{4}}\left(1-\hat{\alpha_{4}}\right){\left[1-2q_{1}q_{2}q_{3}q_{4}\right]}^{-2}/\left(n-1\right)$$ (27)

### 5.2. Efficiency comparison

In this context, our aim is to show the particular circumstances in which the suggested estimator, with four-stage random tool, surpasses estimators that suggested by Warner, Mangat & Singh, and Aboalkhair.

The suggested model is more effective than Warner’s model iff:


$$q_{2}q_{3}q_{4}<\left[1-q_{1}\right]{\left[1-q_{1}q_{2}q_{3}q_{4}\right]}^{-1}$$ (28)

This is achievable by selecting appropriate values for $q_{2}q_{3}q_{4}$ while maintaining a suitable practicable value for $q_{1}$.

**Fig 2:**
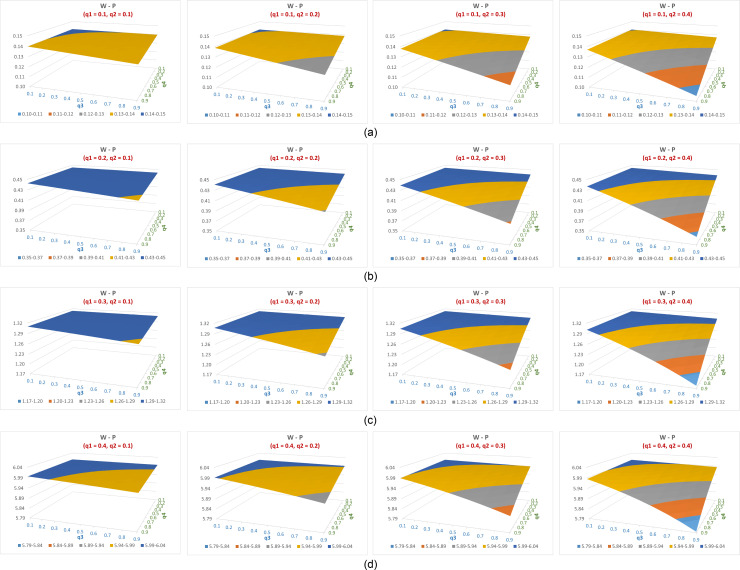
(a–d) The difference in efficiency between the suggested estimator (P) and Warner’s estimator (W) across feasible q_1 values and varying q_(2,) q_3 and 〖 q〗_4 values.

The difference in efficiency between the suggested estimator (P) and Warner’s estimator (W) across feasible $q_{1}$ values and varying $q_{2,}q_{3}$ and $q_{4}$ values is illustrated in Fig 2a–2d. The vertical axis indicates efficiency difference, and the other two axes indicate the values of $q_{3}$ and $q_{4}$. Parts a,b,c,d of the figure are for $q_{1}=0.1,0.2,0.3,0.4$ repectively and for practical values of $q_{2}$ (less than 0.5). Positive values indicate a clear advantage in favor of the suggested estimator in all cases.

It can be noted that (Fig 2a–2d):

1. The estimates from the suggested model exhibit superior efficiency compared to those of Warner’s for $q_{1}<0.5$ and all values of the probabilities $q_{2},q_{3},q_{4}$.
2. When $q_{1}$ increases from 0.1 to 0.4 and fixing $q_{2,}q_{3},q_{4}$, the efficiency difference between the suggested estimate and that of Warner’s also increases.
3. When fixing any set of three out the four probabilities $q_{\text{i}}$$\left(i=1,2,3,4\right)$, the efficiency difference between the suggested estimate and that of Warner’s increases as the fourth probability decreases from 0.9 to 0.1. This is mainly because the fixed efficiency of Warner’s estimate whereas that of the suggested estimate always decreases when any of the $q_{\text{i}}$ decreases.

The suggested model is more effective than Mangat & Singh’s model (MS) if:


$$q_{3}q_{4}<\left[1-q_{1}q_{2}\right]{\left[1-q_{1}q_{2}q_{3q_{4}}\right]}^{-1}$$ (29)

This is achievable by selecting appropriate values for $q_{3}q_{4}$ while maintaining a suitable practicable value for $q_{1}$ and $q_{2}$.

**Fig 3:**
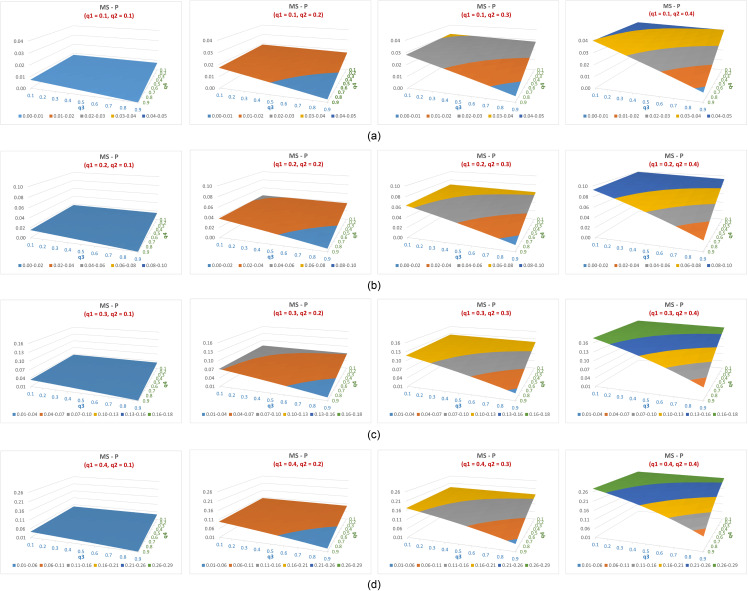
(a–d) The difference in efficiency between the suggested estimator (P) and Mangat & Singh’s estimator (MS) across feasible q_1 values and varying q_(2,) q_3 and 〖 q〗_4 values.

The difference in efficiency between the suggested estimator (P) and Mangat & Singh’s estimator (MS) across feasible $q_{1}$ values and varying $q_{2,}q_{3}$ and $q_{4}$ values is illustrated in Fig 3 (a–d). Same settings as in Fig 2 are used for $q_{1},q_{2,}q_{3},q_{4}$. Positive values indicate the advantage of the suggested estimator in terms of efficiency.

It can be noted that (Fig 3a–3d):

1. The estimates from the suggested model are more efficient than that of MS for all values of $q_{3}$, $q_{4}$ and at practicable values range (0.1 to 0.4) of $q_{1}$,$q_{2}$.
2. When fixing $q_{3}$ and any set of two out the three probabilities $q_{\text{i}}$$\left(i=1,2,4\right)$, the efficiency difference between the suggested estimate and that of MS increases as the fourth probability increases from 0.1 to 0.4.
3. When fixing $q_{1}$,$q_{2}$ and any of the remaining two probabilities $q_{\text{i}}$$\left(i=3,4\right)$, the efficiency difference between the suggested estimate and that of MS increases as the fourth probability decreases from 0.9 to 0.1. This is mainly because the variance of MS estimate is fixed whereas that of the suggested estimate always decreases when any of the $q_{\text{i}}$ decreases.

The suggested estimator is more effective than Aboalkhair’s estimator iff:


$$q_{4}<\left[1-q_{1}q_{2}q_{3}\right]{\left[1-q_{1}q_{2}q_{3}q_{4}\right]}^{-1}$$ (30)

This is achievable by selecting appropriate values for $q_{4}$ while maintaining a suitable practicable value for $q_{1}$,$q_{3}$ and $q_{2}$.

**Fig 4:**
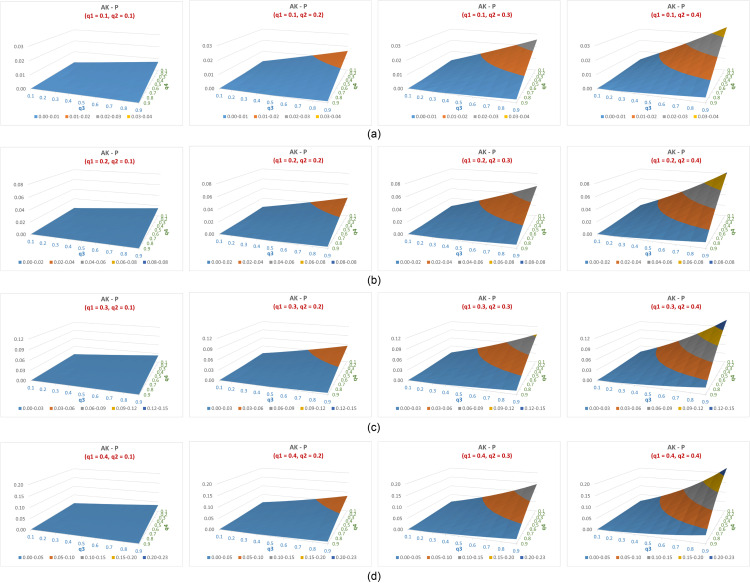
(a–d) The difference in efficiency between the suggested estimator (P) and Aboalkhair’s estimator (AK) across feasible q_1 values and varying q_(2,) q_3 and 〖 q〗_4 values.

The difference in efficiency between the suggested estimator (P) and Aboalkhair’s estimator (AK) across feasible $q_{1}$ values and varying $q_{2,}q_{3}$ and $q_{4}$ values is illustrated in Fig 4a–4d. In this comparison as well, all differences were positive in favor of the proposed estimator.

It can be noted that (Fig 4a–4d):

1. The estimates from the generalized suggested model are more efficient than that of AK for all values of $q_{4}$ and at practicable values range (0.1 to 0.4) of $q_{1}$,$q_{2},q_{3}$.
2. When fixing $q_{4}$ and any set of two out the three probabilities $q_{\text{i}}$$\left(i=1,2,3\right)$, the efficiency difference between the suggested estimate and that of AK increases as the fourth probability increases from 0.1 to 0.4.
3. The efficiency difference between the suggested estimate and that of AK always increases as $q_{4}$ decreases and fixing all other probabilities $q_{\text{i}}$$\left(i=1,2,3\right)$.

### 5.3. Practical guidelines for applying the suggested model in real-world scenarios

To demonstrate the process of estimating the proportion of individuals possessing a sensitive trait using the proposed model in real-world contexts, let’s say with a four-stage random tool, the following practical guidelines are recommended to be followed:

- The survey administrator customs the random device to an optimal number of stages (j), aligning with their assessment of real-world circumstances to strike a balance between efficiency and simplicity (j=4 in this case)
- For the customized random device, the survey administrator sets a probability $p_{5-g}$ for selecting a delicate statement in each stage (g=1,2,3,4) where $p_{5-g}+q_{5-g}=1$.
- A suitable random sample of ‘n’ participants is chosen.
- At the beginning of the trial, a concise overview is provided, outlining the entire process and emphasizing the design’s focus on safeguarding privacy.
- Each participant receives ‘Yes’ and ‘No’ cards, along with a four-stage random device.
- They are instructed to pick a card based on the random device’s outcome and their actual status regarding the sensitive attribute.
- Depending on the random device’s result, the process may end at any stage (g = 1,2,3,4).
- Participants discreetly place their chosen card into a container without disclosing to the interviewer their selection or at which stage the process has ended.
- The estimation of the proportion of individuals with a sensitive trait and its variance is accomplished by analyzing the sample outcomes and utilizing Eqs. (24 and 27).

## 6. Discussion

Comparisons of efficiency indicate that the suggested RR model with a four-stage random tool offers a more effective substitute for all of Warner [1], Mangat & Singh [5], and Aboalkhair [20] models. Furthermore, Aboalkhair’s model proves to be a more effective substitute for models suggested by Warner and Mangat & Singh. Similarly, Mangat & Singh’s model offers a more effective substitute for Warner’s model.

Setting value 0.1 for each of the probabilities $q_{1},q_{2},q_{3},q_{4}$ appears to be the most efficient, and the least favorable, in laying the foundation of privacy protection. On the other hand, set value 0.9 for each of the probabilities $q_{1},q_{2},q_{3},q_{4}$ appears as the optimal value for protecting privacy as it is the lowest value for efficiency. Hence, opting for any of the combinations 0.5, 0.5, 0.5, 0.8; 0.5, 0.5, 0.8, 0.5; 0.5, 0.8, 0.5, 0.5; or 0.8, 0.5, 0.5, 0.5 as values for the probabilities $q_{1},q_{2},q_{3}$ and $q_{4}$ is quite rational. This selection will make sure that both the privacy and efficiency of the suggested model are comparable to the model that suggested by Warner when $p_{1}=0.9$. Furthermore, it is more likely to achieve the desirable result of targeting specific questions containing sensitive information without making interviewees too suspicious and to contribute to their cooperation.

**Fig 5:**
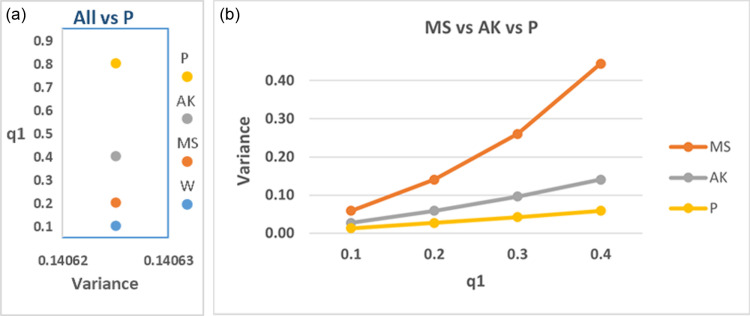
(a, b) The variances of the proposed estimator, Aboalkhair’s estimator, Mangat & Singh’s estimator, and Warner’s estimator at selected values for q_1, q_2, q_3, q_(4).

From Fig 5, it can be deduced that the suggested model exhibits superior efficiency compared to those of Mangat & Singh [5] and Aboalkhair [20] when $q_{2}=q_{3}=q_{4}=0.5$, regardless of the value of $q_{1}$. Also, when $q_{2}=q_{3}=q_{4}=0.5$ and $q_{1}=0.8$, the suggested model efficiency equivalent to the efficiency of Warner’s model at $q_{1}=0.1$, Mangat & Singh’s model at $q_{1}=0.2$, and Aboalkhair’s model at $q_{1}=0.4$. These outcomes confirm the core idea of the generalized suggested model, which aims at increasing efficiency, suggesting the utilization of an increasing number of random devices while assigning low probability for choosing the sensitive question.

## 7. Limitations and future research

A possible limitation of the suggested model is that it views a general framework for some earlier models and generator of new efficient models in situations where complete honesty is expected. However, when it comes to highly delicate matters, then the probability of incomplete truthfulness arises. Which in turn opens up a future avenue to revise this model to comply with a scenario of incomplete truthfulness, and hence make it more suitable for accurately determining extremely sensitive characteristics.

## Supporting information

S1 File Data file. (XLSX)

S2 File Data set for Figure 2. (XLSX)

S3 File Data set for Figure 3. (XLSX)

S4 File Data set for Figure 4. (XLSX)

S5 File Data set for Figure 5. (XLSX)
